# Gadolinium(III) Complexes with N-Alkyl-N-methylglucamine Surfactants Incorporated into Liposomes as Potential MRI Contrast Agents

**DOI:** 10.1155/2015/942147

**Published:** 2015-08-10

**Authors:** Simone Rodrigues Silva, Érica Correia Duarte, Guilherme Santos Ramos, Flávio Vinícius Crizóstomo Kock, Fabiana Diuk Andrade, Frédéric Frézard, Luiz Alberto Colnago, Cynthia Demicheli

**Affiliations:** ^1^Departamento de Química, Instituto de Ciências Exatas, Universidade Federal de Minas Gerais, 31270-901 Belo Horizonte, MG, Brazil; ^2^Departamento de Fisiologia e Biofísica, Instituto de Ciências Biológicas, Universidade Federal de Minas Gerais, 31270-901 Belo Horizonte, MG, Brazil; ^3^Embrapa Instrumentação, Empresa Brasileira de Pesquisa Agropecuária, 13560-970 São Carlos, SP, Brazil

## Abstract

Complexes of gadolinium(III) with N-octanoyl-N-methylglucamine (L8) and N-decanoyl-N-methylglucamine (L10) with 1 : 2 stoichiometry were synthesized and characterized by elemental analysis, electrospray ionization-tandem mass spectrometry (ESI-MS), infrared (IR) spectroscopy, and molar conductivity measurements. The transverse (*r*_2_) and longitudinal (*r*_1_) relaxivity protons were measured at 20 MHz and compared with those of the commercial contrasts. These complexes were incorporated in liposomes, resulting in the increase of the vesicle zeta potential. Both the free and liposome-incorporated gadolinium complexes showed high relaxation effectiveness, compared to commercial contrast agent gadopentetate dimeglumine (Magnevist). The high relaxivity of these complexes was attributed to the molecular rotation that occurs more slowly, because of the elevated molecular weight and incorporation in liposomes. The results establish that these paramagnetic complexes are highly potent contrast agents, making them excellent candidates for various applications in molecular MR imaging.

## 1. Introduction

Magnetic resonance imaging (MRI) is one of the most powerful noninvasive techniques that yields high quality anatomical and functional imaging of the human or animal bodies [1–3]. The acquisition of images of soft tissue by MR occurs mainly by the use of contrast agents, which correspond to 35% of the diagnoses. The most common contrast agents (CAs) are Gd(III)-based complexes [4].

The diagnostic is based on image contrast between healthy and abnormal tissues or organs. Three common contrast methods are based on proton density (PD) and longitudinal (*T*_1_) and transverse (*T*_2_) relaxation times [5]. In the relaxation weight images known as *T*_1_- and *T*_2_-weighted images, the contrast depends on *T*_1_ and *T*_2_, respectively [6]. The Gd(III)-based CAs, commonly referred to as *T*_1_-agents, reduce the longitudinal relaxation time and increase the positive contrast in *T*_1_-weighted image [7, 8].

Gd(III) ion has a high magnetic moment due to the seven unpaired electrons. Moreover, the high relaxivity of these CAs depends on the number of coordinated water molecules (*q*) and the correlation time (*τ*_*R*_). The correlation time depends on the molecular rotational correlation times (*τ*_*R*_) of the complex, the exchange rate (*τ*_*M*_) of the coordinated water molecules, and the electronic relaxation time of the metal ion (*τ*_*e*_) [9].

In the Gd(III)-based CAs, the coordinating ligands leave one or two free sites for water coordination in the Gd(III) ion [10]. Water molecules that are coordinated to the metal center give a direct contribution to relaxivity, while the bulk solvent molecules experience the paramagnetic effect when they diffuse around the metal center. Those are the two main interactions that contribute to the observed relaxivity, known as the inner-sphere relaxation rate and outer-sphere relaxation rate, respectively [11].

The most important, classical, and clinically used *T*_1_-agents are gadopentetate dimeglumine (Magnevist), gadoterate meglumine (Dotarem), gadoteridol (ProHance), and gadodiamide (Omniscan) [12]. They have molecular weight in the range of 600–700 Da and relaxivities between 4 and 5 mM^−1^ s^−1^ at 20 MHz and 310 K. These agents are used to delineate lesions in the brain as a result of disruption of the blood-brain barrier. Two derivatives of Gd-diethylenetriaminepentaacetic acid were introduced recently in clinical use, gadobenate dimeglumine (MultiHance) and gadoxetate disodium (Eovist) [13]. These compounds are more lipophilic than the classical agents and have affinity towards human serum and albumin, being specifically accumulated in hepatocytes [14].

The actual strategies to design new highly sensitive *T*_1_-agents are mainly based on two approaches [15, 16]: (i) slowing down the molecular rotation by increasing the molecular weight or by binding Gd(III) complexes to systems of different dimensions and (ii) increasing the number of Gd(III) complexes loaded on a suitable carrier. In this approach, nanotechnology offers a wide portfolio of nanocarriers including dendrimers, micelles, liposomes, solid lipid nanoparticles, nanoemulsions, and other nanosystems [17].

In this context, the liposomes have technically several advantages: (1) high biocompatibility; (2) easiness of preparation; (3) great chemical versatility (ability to be loaded with hydrophobic, amphiphilic, and hydrophilic substances); (4) simplicity of decorating the surface with targeting ligands, blood lifetime modulators, drugs, diagnostic tracers, and so forth; and (5) a longstanding and well-established clinical use as drug-delivery carriers [15, 18].

Several different amphiphilic Gd(III) complexes have been investigated for this purpose [19–22], with emphasis on two types of ligands: (1) diethylenetriaminepentaacetic acid (DTPA) and (2) macrocyclic 1,4,7,10-tetraazacyclododecane-1,4,7,10-tetraacetic acid (DOTA) [23, 24]. Recently, new amphiphilic Gd-DOTA-like complexes with two aliphatic chains in the same paramagnetic center have been investigated. The presence of the aliphatic chains on adjacent coordinating arms was shown to be a good strategy to design liposomal MRI *T*_1_-agents [15, 25].

**Figure 1:**
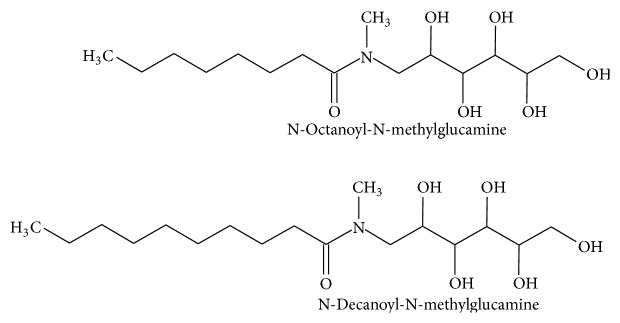
The chemical structure of amphiphilic ligands N-octanoyl-N-methylglucamine (L8) and N-decanoyl-N-methylglucamine (L10) used in this work for complexation with Gd(III).

In this work, complexes of Gd(III) with aliphatic chain ligands of N-alkyl-N-methylglucamine series (Figure 1) were synthesized with a stoichiometric ratio of 1 : 2 metal : ligands. The presence of two aliphatic chains on adjacent coordinating arms was conceived to reduce the local rotational motion of the Gd(III) complexes after incorporation in the liposomal bilayer.

The complexes were characterized by elemental analysis, infrared (IR) spectroscopy, electrospray ionization-tandem mass spectrometry (ESI-MS), and molar conductivity measurements. The complexes were incorporated into liposomes and their particle size and zeta potential were investigated by Zetasizer. The relaxivity measurements (*r*_1_ and *r*_2_) were evaluated for Gd(III) complexes in free form and those incorporated in liposomes.

## 2. Materials and Methods

### 2.1. Materials and Drugs

N-Octanoyl-N-methylglucamine (L8; 98%), N-decanoyl-N-methylglucamine (L10; 98%), gadolinium chloride hexahydrate GdCl_3_·6H_2_O, and 1,6-diphenylhexatriene (DPH) were obtained from Sigma Aldrich Chemical Co. (USA). Soybean phosphatidylcholine (SPC, Phospholipon 90) was obtained from Lipoid (Germany). Magnevist was obtained from Bayer (Germany).

### 2.2. Physical Measurements

Infrared spectra were recorded on a Perkin-Elmer Spectrum FT-IR spectrometer in the range 4000–400 cm^−1^ using KBr pellets. ESI-MS spectra were collected on a Thermo-Scientific LCQ Fleet mass spectrometer operating in positive mode. To obtain the spectra, samples were dissolved in methanol and were injected in the apparatus by direct infusion with a 10 *μ*L min^−1^ flow, using the following main instrument parameters: capillary voltage 2.5 kV and cone voltage 25 kV. CHN microanalyses were carried out using Perkin-Elmer 2400 C, H, and N elemental analyses. Conductivity measurements were performed using Digimed DM 31 in a cell at 25°C in DMF at a complex concentration of approximately 1 mM.

The lipophilic fluorescent probe DPH was used to identify hydrophobic microenvironment and determine the critical micelle concentration (CMC). The method described by Fernandes and coworkers was used [26]. Fluorescence measurements were carried out using a Cary Eclipse fluorescence spectrometer (Varian Inc.). Temperature was controlled at 25°C through a jacketed cuvette holder from a refrigerated circulating water bath.

The NMR measurements were performed in a 0.5 T Bruker minispec mq20 low-resolution NMR spectrometer (^1^H 19.9 MHz) equipped with a 10 mm wide commercial temperature range probe. *T*_1_ and *T*_2_ measurements were performed using Inversion-Recovery (IR) and Carr-Purcell-Meiboom-Gill (CPMG) pulse sequences, respectively, using *π*/2 = 3.1 *μ*s and *π* = 6.2 *μ*s. *T*_1_ measurements were performed using inversion time from 10 to 20000 ms, and *T*_2_ measurements were performed using echo time of 2 ms and 5000 echoes and four scans with a repetition time of 15 s. Four concentrations of 0.1, 0.2, 0.5, and 1 mM of the free complexes were prepared in aqueous solution. For the complexes incorporated in liposomes, the relaxivity was measured in 0.3 M sucrose at lipid concentration of 0.5, 1.0, and 1.5 mM.

### 2.3. Synthesis of the Complexes

A general synthetic route was used in which a sample of GdCl_3_·6H_2_O (0.16 g, 0.25 mmol) was dissolved in 20.0 mL of deionized water followed by the addition of the appropriate ligand (0.5 mmol) to the solution. The resulting solution was stirred at 60°C until complete evaporation of the solvent. Acetone was added to yield a precipitate, which was filtered, washed with acetone, and dried under vacuum, yielding a white powder.

#### 2.3.1. GdL8 Complex

Yield: 70%; Melting point: 210°C (dec.); IR (KBr, cm^−1^): 3350 (*ν*-OH), 1614 (*ν*-CO), 1080 (*ν*-CN), 616 (*δ*-NCO); ESI-MS:* m/z* 798.08 [Gd(OCT)_2_]^+^;* Anal*.* Calc*. for C_30_H_60_N_2_O_12_GdCl (%) (Mr = 833.51): C, 43.06; H, 7.99; N, 3.57; Found: C, 43.23; H, 7.26; N, 3.36. The molar conductance in DMF was 66.05 Λ_m_ (Ω^−1^ cm^2^ mol^−1^).

#### 2.3.2. GdL10 Complex

Yield: 85%, Melting point: 227°C (dec.); IR (KBr, cm^−1^): 3350 (*ν*-OH), 1602 (*ν*-CO), 1078 (*ν*-CN), 618 (*δ*-NCO); ESI-MS:* m/z* 854.23 [Gd(DEC)_2_]^+^:* Anal*.* Calc*. For C_34_H_78_N_2_O_17_GdCl (%) (Mr = 979.69): C, 41.31; H, 8.32; N, 2.58; Found: C, 41.68; H, 8.02; N, 2.86. The molar conductance in DMF was 67.56 Λ_m_ (Ω^−1^ cm^2^ mol^−1^).

### 2.4. Liposome Preparation and Characterization

Liposomes were prepared by the thin-film method as follows: Soybean phosphatidylcholine (SPC) and Gd(III) complex were first codissolved in chloroform at a 10 : 1 SPC : complex molar ratio and solvent was evaporated to dryness under vacuum. The thin film obtained was hydrated with a 0.3 M sucrose solution at a final lipid concentration of 15 mM. The liposome suspensions were then repeatedly (5 times) extruded at 25°C through 200 nm polycarbonate membrane.

The particle mean hydrodynamic diameter, polydispersity index (PDI), and zeta potential were determined, after dilution of the dispersion in 0.3 M sucrose at 0.075 mM lipid concentration, by dynamic light scattering using a Zetasizer (Nano ZS90, Malvern Instruments, United Kingdom). Dispersion Technology Software, version 6.12, was used to collect and analyze the data. The samples were kept at 25°C during the entire experiments and analyzed at a fixed angle of 90°.

## 3. Results and Discussion

### 3.1. Characterization of Gd(III) Complexes

**Figure 2:**
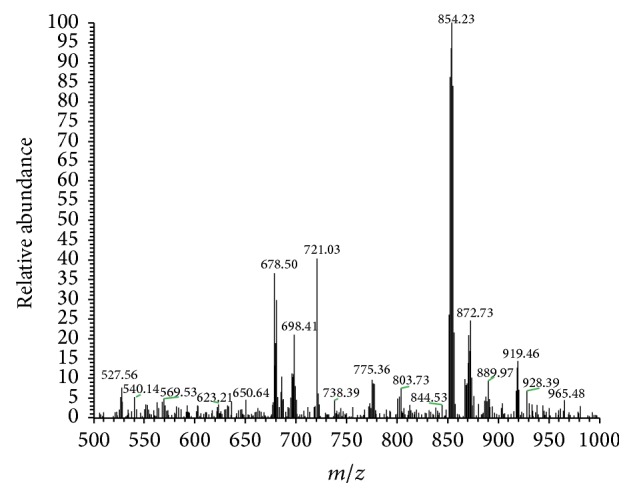
ESI-MS spectrum of GdL10 in the positive mode.

The data from elemental and thermogravimetry analyses of the synthesized complexes indicate that 1 : 2 stoichiometry and conductivity values suggest 1 : 1 electrolytes. ESI-MS data also supports the formation of 1 : 2 Gd-ligand complexes, with deprotonation of a hydroxyl in each ligand molecule.  Figure 2 presents the ESI-MS spectrum of GdL10 in the positive mode, and Table 1 displays the main species identified in GdL8 and GdL10 and their proposed structures. Accordingly, the main peak was attributed to 1 : 2 Gd-ligand complexes.

Hence, the data obtained were in agreement with the formulae [Gd(OCT)_2_]Cl (GdL8) and [Gd(DEC)_2_]Cl·5H_2_O (GdL10). The inclusion of a drying step (heating and solvent evaporation) in the synthetic process also greatly favors the formation of the complexes, in spite of the high p*K*_*a*_ values of the hydroxyl groups (p*K*_*a*_ ~ 12).

IR spectra show that the region corresponding to *ν*(C=O) at 1632 cm^−1^ for free ligand shifted to lower frequencies and was observed for gadolinium compounds at 1614 cm^−1^. The shift of the carbonyl peak for smaller wave number indicates the involvement of this group in the formation of metal complex. The peak at 1098 cm^−1^ is attributed to the *ν*(C–OH). The shift of the band from 616 cm^−1^ to 632 cm^−1^ for the gadolinium complex is attributed to the *ν*(N–C=O); this angular deformation in the plane suggests that the amide group of the ligand also coordinates to the metal. Changes in the region from 400 to 500 cm^−1^ for the Gd(III) complexes are assigned to the appearance of characteristic bands of metal-oxygen.

**Figure 3:**
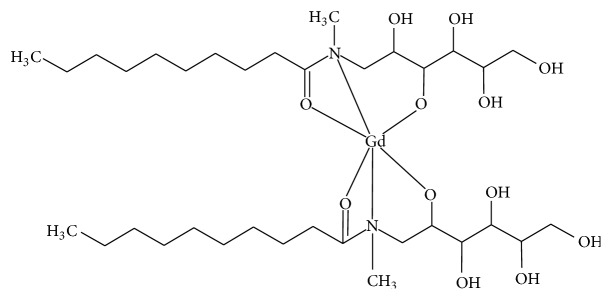
Structure of the Gd(III) complex with N-alkyl-N-methylglucamine ligand.

This data taken altogether suggests that Gd(III) is hexacoordinated to two molecules of the asymmetric tridentate ligands, through the nitrogen of the amine, the oxygen atom of the carbonyl group, and the deprotonated oxygen of the ligand. Nevertheless, since Gd^3+^ ions prefer coordination number of 8-9, the central metal ion may also bind at least two water molecules to complete the sphere of coordination in aqueous solution. All attempts to obtain monocrystal of the complexes were unsuccessful. Thus, no information about different possible diastereoisomers could be obtained.  Figure 3 shows the structure of one possible diastereoisomer without considering the bound water molecules.

The lipophilic DPH probe shows marked fluorescence increase, when transferred from water to hydrophobic microenvironment, a property that has been exploited to estimate the CMC of surfactants [27]. When incubated in the presence of L8 and L10 dispersions in water, the DPH probe exhibited an increase in fluorescence intensity, from the surfactant concentration of about 50 and 3 mM, respectively. Interestingly, the dispersions of GdL8 and GdL10 complexes showed the formation of hydrophobic environments at lower concentrations of L8 and L10, about 6 and 2 mM, respectively. This data suggests that complexation with Gd(III) enhances the thermodynamic stability of the surfactant nanoassemblies.

The complexes were found to be stable for at least two days upon storage at room temperature in diluted aqueous solution (0.1 mM). This long-term stability was evidenced by UV-Vis experiments showing no change in the spectra of the complexes as a function of time (data not shown). This stability data supports the potential of these complexes as contrast agents.

**Figure 4:**
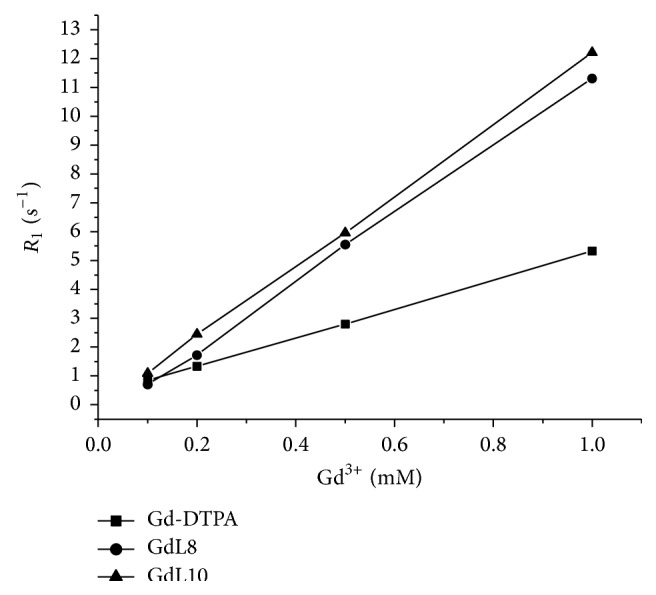
Correlation curves between the longitudinal relaxation rates (*R*_1_) of GdL8 and GdL10 and Gd-DTPA in aqueous solution, as a function of concentration, at 25°C and 0.47 T (20 MHz).

**Figure 5:**
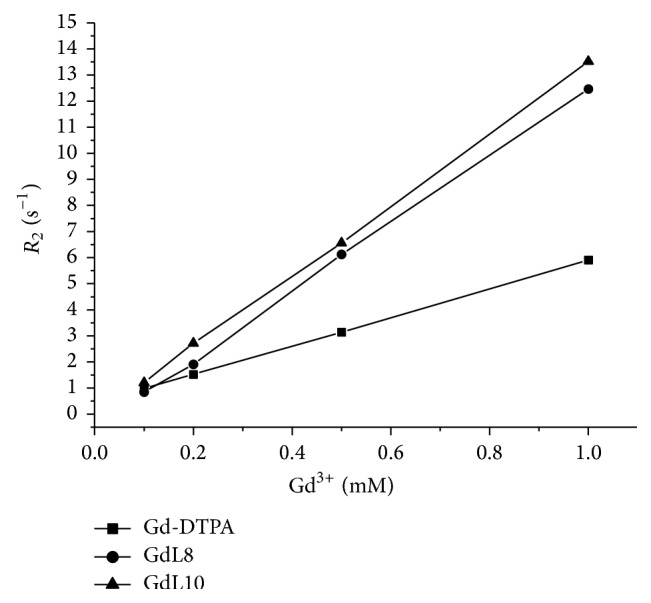
Correlation curves between the transverse relaxation rates (*R*_2_) of GdL8 and GdL10 and Gd-DTPA in aqueous solution, as a function of concentration, at 25°C and 0.47 T (20 MHz).

Figures 4 and 5 show the curves between the longitudinal (1/*T*_1_ = *R*_1_) and transverse relaxation rate (1/*T*_2_ = *R*_2_) of GdL8 and GdL10 and of the commercial contrast agent dimeglumine gadopentetate (Gd-DTPA) in aqueous solution, as a function of concentration, at 25°C and 0.47 T (20 MHz).

The slopes of the curves of Figures 4 and 5 calculated by linear fitting give the longitudinal (*r*_1_) and transverse (*r*_2_) relaxivity, respectively, and are shown in Table 2.

Thus, GdL8 and GdL10 compounds exhibit higher relaxivity (*r*_1_ ~ *r*_2_ > 12 s^−1^ mM^−1^) than commercial contrast agents DTPA with *r*_1_ ~ *r*_2_ ~ 5 s^−1^ mM^−1^. According to the literature [28], the longitudinal relaxivity values of commercial contrast agents range from 3 to 5 s^−1^ mM^−1^.

In the present work, the amphiphilic Gd(III) complexes also show values of relaxivity nearly twice higher than those reported by Ratnakar et al. for Gd(III) complex of glucose-6-phosphate conjugated to 1,4,7,10-tetraazacyclododecane-1,4,7-triacetic acid [29] and by Zhang et al. for tetranuclear macrocyclic complexes of Gd(III) (*r*_1_ ~ 7.0 s^−1^ mM^−1^) [4].

The closest relaxivity values found in the literature are those reported for Gd(III) complexes of polymeric micelles and the amphiphilic gadolinium complex (C18)_2_ DTPA (Gd) in monoolein and diolein (*r*_1_ ~ 10.0–13.0 s^−1^ mM^−1^) [30, 31].

In other studies, Othman et al. and Vaccaro et al. synthesized and characterized amphiphilic complex obtained by coupling the hydrophilic DOTA ligand [1,4,7,10-tetrakis(carboxymethyl)-1,4,7,10-tetraazacyclododecane] to squalenoyl moieties and complexes containing amphiphilic supramolecular aggregates DTPAGlu chelating agent covalently bound to two C18 alkylic chains with a good relaxivity of 15–22 s^-1 ^mM^−1^ (at 20 MHz and 37°C) and 21.5–24 s^-1 ^mM^−1^ (at 20 MHz and 25°C), respectively [32, 33].

The increase of the relaxation can be explained by the rotational correlation time caused by the molecular weight or the formation of aggregates of the amphiphilic gadolinium complexes giving a further increase in *τ*_*R*_ [34]. Considering that the relaxation measurements of GdL8 and GdL10 dispersions were performed below the CMC, their high relaxivities most probably arise from their high molecular weight and the presence of two free positions for water coordination, rather than their ability to self-associate into nanoassemblies.

### 3.2. Effect of Incorporation of Gd(III) Complexes into Liposomes

The Lipo-GdL8 and Lipo-GdL10 compounds were incorporated into phosphatidylcholine liposomes, as an attempt to further enhance the relaxivity.  Table 3 displays the physical properties of the liposomes in the absence or in the presence of the different complexes. These properties include the mean particle diameter, the polydispersity index, and zeta potential. The particle sizes of the liposomes incorporating Lipo-GdL8 complex were slightly lower than those of empty liposomes and those containing Lipo-GdL10. As shown in Table 3, the zeta potential of liposomes changed from negative to positive values upon incorporation of the Gd(III) complexes. This data is consistent with the positive charge of the complexes and their incorporation and localization at the solution-membrane interface. The highest zeta potential value was obtained for Lipo-GdL8 followed by Lipo-GdL10.

**Figure 6:**
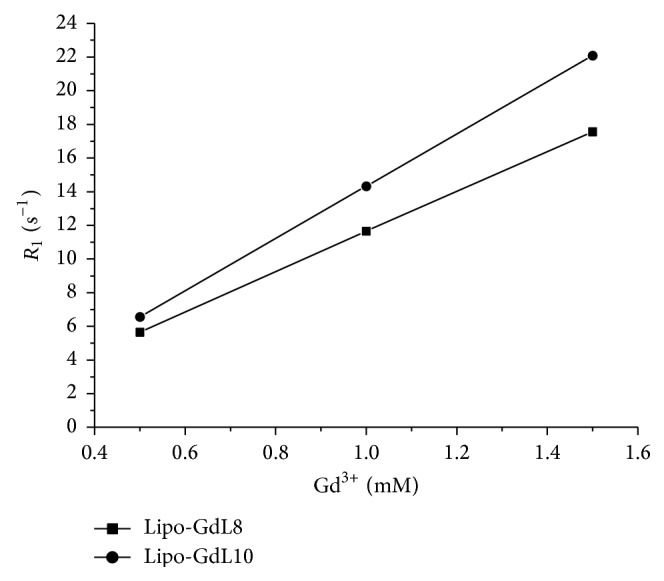
Correlation curves between the longitudinal relaxation rates (1/*T*_1_ = *R*_1_) of Lipo-GdL8 and Lipo-GdL10 incorporated in liposomes at different concentrations of Gd(III).

**Figure 7:**
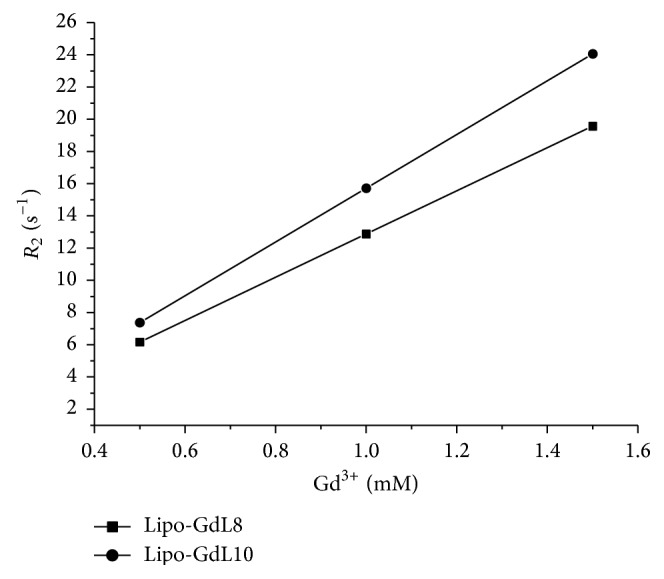
Correlation curves between the transverse relaxation rates (1/*T*_2_ = *R*_2_) of Lipo-GdL8 and Lipo-GdL10 incorporated in liposomes at different concentrations of Gd(III).

Figures 6 and 7 show the curves between the longitudinal (1/*T*_1_ = *R*_1_) and transverse relaxation rate (1/*T*_2_ = *R*_2_) of Lipo-GdL8 and Lipo-GdL10 incorporated in liposome at different concentrations (0.5, 1.0, and 1.5 mM) at 25°C and 0.47 T (20 MHz).

Table 4 shows the relaxivity values (*r*_1_ and *r*_2_, in units of s^−1^ mM^−1^) of the complexes, as determined from the slopes of linear regression of the curves shown in Figures 6 and 7.

Incorporation into liposomes did not increase the relaxivity of Lipo-GdL8 complex (*r*_1_ ~ 11.92 s^−1^ mM^−1^). However, an increase in relaxivity was observed for Lipo-GdL10 incorporated in liposomes. *r*_1_ and *r*_2_ relaxivities of the compound increase from approximately 12.3 to 15.5 and from 13.6 to 16.7 s^−1^ mM^−1^. Moreover, the relaxivity values observed with complexes liposome incorporation are about three times higher than those of the commercial contrast agents used clinically (*r*_1_ ~ 3.0–5.0 s^−1^ mM^−1^).

The fact that relaxivity of GdL8 complex was unaffected by incorporation into liposomes may be due to the reduced vesicle diameter (141 nm), resulting in elevated relaxation times, or to little restriction of the rotational flexibility of the complex. In addition to the greater diameter of the vesicle (169 nm) of Lipo-GdL10 sample, other factors may contribute to the significant increase of GdL10 relaxivity upon liposome incorporation. It is known that the incorporation of metal compound into nanoparticles can increase *r*_1_ due to the restriction of rotational flexibility of the compounds. Furthermore, the exposition of the metal at the external aqueous surface of the particle may facilitate the interaction of water molecules with the paramagnetic center [35]. In a recent work, Cittadino et al. [15] investigated MRI performance of liposome incorporating either complex Lipo-GdDOTA-(GAC_12_)_2_ or Lipo-GdDOTAMA-(GAC_18_)_2_. The presence of two aliphatic chains on adjacent coordinating arms was found to reduce considerably the rotational motion of the Gd(III) chelates incorporated in the liposome bilayer, increasing the relaxivity of the complex. From this perspective, one can propose a similar reduction of rotational flexibility in the case of Lipo-GdL10, where the aliphatic chains may be positioned on the nanoparticle like tweezers.

Kozlowska et al. [36] studied polychelating amphiphilic polymers with Gd(III) incorporated into liposomes. At low frequency (15–25 MHz), the values of *r*_1_ were in the range of 13-14 s^−1^ mM^−1^, values close to that found for the complex. Kielar et al. [25] showed that gadolinium compounds (Gd-DOTA(GAC_12_) and Gd-DOTA(GAC_12_)_2_) in liposomes are good strategy to improve the relaxivity with 17 and 40 s^−1^ mM^−1^, respectively.

One expected benefit of the incorporation of these amphiphilic complexes in liposomes is the reduction of their toxicity, as exploited in the case of several amphiphilic drugs commercialized under the liposomal form [37]. Furthermore, liposomes characteristics can be manipulated so as to achieve either passive or active targeting to a specific tissue and improved RMI contrast agents [38].

## 4. Conclusions

The complexes GdL8 and GdL10 were prepared and characterized by elemental analyses, IR, mass spectrometry, CMC, and relaxometry measurements. The gadolinium complexes have 1 : 2 stoichiometry, confirmed by elemental analysis and mass spectroscopy. The incorporation of the Gd(III) complexes in liposomes was accompanied by an increase of the vesicle zeta potential. Both the free and liposome-incorporated gadolinium complexes showed high relaxation effectiveness, compared to commercial contrast agent Gd-DTPA, presumably because of the molecular rotation that occurs more slowly because of the elevated molecular weight and incorporation in liposomes. The results showed that both of these paramagnetic complexes are highly potent contrast agents, making them excellent candidates for various applications in molecular MR imaging.

## Figures and Tables

**Table 1:** ESI-MS characterization of GdL8 and GdL10 complexes.

Cationic species	*m*/*z*	
	GdL8	GdL10
L + H^+^	322.13	
[Gd(L)_2_]^+^	^a^798.08	^a^854.23
[Gd(L)_2_]^+^ + H_2_O		872.73
[Gd(L)_2_]Cl − e^−^	833.88	889.97

^a^Peak of higher intensity.

**Table 2:** *r*_1_ and *r*_2_ relaxivity of Gd complexes at 0.47 T (25°C).

Compound	*r*_1_ (s^−1^ mM^−1^)	*r*_2_ (s^−1^ mM^−1^)
Gd-DTPA	4.98 ± 0.03	5.47 ± 0.03
GdL8	11.90 ± 0.02	13.00 ± 0.02
GdL10	12.30 ± 0.01	13.60 ± 0.02

**Table 3:** Physical properties of liposomes (mean diameter, PDI, and zeta potential).

Complex	Diameter (nm)	PDI	*Z* potential (mV)
No complex	166.3	0.133	−25.4
Lipo-GdL8	141.0	0.197	14.7
Lipo-GdL10	169.4	0.083	5.4

**Table 4:** *r*_1_ and *r*_2_ relaxivity of the Gd(III) complexes incorporated in liposome at 0.47 T (25°C).

Complex	*r*_1_ (s^−1^ mM^−1^)	*r*_2_ (s^−1^ mM^−1^)
No complex	—	—
Lipo-GdL8	11.92 ± 0.03	13.41 ± 0.05
Lipo-GdL10	15.53 ± 0.12	16.68 ± 0.09
